# Comprehensive circular RNA profiling reveals that circular RNA100783 is involved in chronic CD28-associated CD8(+)T cell ageing

**DOI:** 10.1186/s12979-015-0042-z

**Published:** 2015-10-08

**Authors:** Yu-hong Wang, Xu-hui Yu, Shan-shun Luo, Hui Han

**Affiliations:** Department of Geriatrics, First Affiliated Hospital of Harbin Medical University, Harbin, 15001 China; First Institute of Geriatrics and Gerontology of Harbin Medical University, Harbin, 15001 China; Department of Ophthalmology, First Affiliated Hospital of Harbin Medical University, Harbin, 15001 China

**Keywords:** T cell, Ageing, CD28, Circular RNA, Microarray, Biomathematics

## Abstract

**Background:**

Ageing brings about the gradual deterioration of the immune system, also known as immunosenescence. The role of non-coding circular RNA in immunosenescence is under studied. Using circular RNA microarray data, we assembled Comparison groups (C1, C2, C3 and C4) that allowed us to compare the circular RNA expression profiles between CD28(+)CD8(+) T cells and CD28(-)CD8(+) T cells isolated from healthy elderly or adult control subjects. Using a step-wise biomathematical strategy, the differentially-expressed circRNAs were identified in C1 (CD28(+)CD8(+) *vs* CD28(-)CD8(+)T cells in the elderly) and C4 (CD28(-)CD8(+)T cells in the elderly *vs* in the adult), and the commonly-expressed circRNA species from these profiles were optimized as immunosenescence biomarkers.

**Results:**

Four overlapping upregulated circular RNAs (100550, 100783, 101328 and 102592) expressed in cross-comparison between C1 and C4 were validated using quantitative polymerase chain reaction. Of these, only circular RNA100783 exhibited significant validation. None of the down-regulated circular RNAs were expressed in the C1 and the C4 cross-comparisons. Therefore, we further predicted circular RNA100783-targeted miRNA-gene interactions using online DAVID annotation. The analysis revealed that a circular RNA100783-targeted miRNA-mRNA network may be involved in alternative splicing, the production of splice variants, and in the regulation of phosphoprotein expression. Considering the hypothesis of splicing-related biogenesis of circRNAs, we propose that circular RNA100783 may play a role in phosphoprotein-associated functions duringCD28-related CD8(+) T cell ageing.

**Conclusions:**

This study is the first to employ circular RNA profiling to investigate circular RNA-micro RNA interactions in ageing human CD8(+)T cell populations and the accompanying loss of CD28 expression. The overlapping expression of circular RNA100783 may represent a novel biomarker for the longitudinal tracking ofCD28-related CD8(+) T cell ageing and global immunosenescence.

**Electronic supplementary material:**

The online version of this article (doi:10.1186/s12979-015-0042-z) contains supplementary material, which is available to authorized users.

## Background

The ongoing loss of immune function during ageing, or immunosenescence, is a major contributor to the increasing morbidity and mortality in the elderly population. One of the prominent alterations on immune cells during this process is the gradual loss of the co-stimulatory molecule, CD28, from the surface of CD8(+) T lymphocytes [1, 2]. Thus, an increase in the CD28(-)CD8(+) T subset is considered to be one hallmark of immunosenescence [3]. However, except for their replicative characteristics during senescence [4, 5], very little is known regarding the exact nature, origin and function of CD28(-)CD8(+)T cells [1]. Indeed, the mechanism by which CD28 is lost from CD28(+)CD8(+)T cell surface is complicated. During an individual’s lifespan, CD28(+)CD8(+)T cells are gradually stimulated by encounters with numerous antigens resulting in CD28 exhaustion. Latent human cytomegalovirus (CMV) is responsible for the greatest clonal expansion of CD28(-)CD8(+) T cells in the healthy elderly population [6–9]. Latent CMV carriers are so prevalent that CMV seropositivity has been proposed as one of the “Immune Risk Profiles (IRPs)”, and individuals in the general elderly population who exhibit high IRP have an increased risk of all-cause mortality [10, 11]. Moreover, microarray analyses of elderly and adult control subjects revealed that 754 genes representing a wide range of biological processes and cellular functions are significantly altered in human CD8(+) T cells [12]. It has been observed that CD28(-)CD8(+) T cells demonstrated similar patterns of gene expression in elderly and adult subjects [13, 14], while the profiles of gene expression in CD28(+)CD8(+) T cells are profoundly altered in adult and the elderly populations [14]. However, the disparities between adult and old, as well as between CD28(-)CD8(+) and CD28(+)CD8(+)T cells, remain unexplored.

More than 25,000 species of an enigmatic class of circular RNAs have been identified in various human cells [15]. At least two circular RNAs have been demonstrated to function as molecular sponges to their target miRNAs [16, 17]. It has been hypothesized that circular RNAs are produced by non-random back-splice events that forma covalently closed continuous loop [18], and some generalized mechanism might strictly determine whether splicing produces a circular non-coding RNA or a linear mRNA [19]. The expression of circRNA was found to be tissue- as well as developmentally-specific [17, 20]. The levels of circular RNAs also increase in an age-related manner in *Drosophila* [21]. Until the present study, age-related circular RNA profiling in lymphocytes, and the circRNA-dependent molecular regulation ofCD28 exhaustion from the surface of CD8(+)T cell, has remained under studied.

The development of circRNA microarray (http://www.kangchen.com.cn/english/) has facilitated the study of the role of circular RNAs in regulating gene expression through a circRNA-miRNA-mRNA network. To explore the underlying molecular regulation of circRNAs in T cell ageing, we first used circular RNA microarray detection to acquire circRNA profiles in CD28-dependent CD8(+)T cell subsets in elderly individuals(Q1 and Q2) and adult controls (Q3 and Q4). Based on it, we compared cross them to acquire four cross-differentiated circular RNA profiles (C1,C2, C3 and C4). Then we performed step-wise bioinformatics analysis to identify the overlapping circular RNA molecules between C1 and C4 as potential biomarkers of CD8+T cell immunosenescence.

## Results and discussions

### Clinical characteristics of the enrolled subjects

We enrolled in our study a total of 21 apparently healthy elderly individuals and 8 healthy adult volunteers, who had been initially identified as CMV latent carriers by CMV-IgG detection. The general clinical characteristics of the 29 participants are shown in Table 1.

**Table 1:** The clinical characteristics of the subjects with CMV seropositivity

Category	Elderly group			Adult group
	Old-old	Middle-old	Adult-old	
Number	6	7	8	8
Age	90.5 ± 2.4	79.6 ± 2.5	69.3 ± 3.1	32.0 ± 4.6
Gender (male vs female)	3vs3	4vs3	4vs4	4vs4
Number of chronic diseases^a^	3.4	3.0	2.9	0
Number of daily medications(NNTs)^b^	5.6	5.3	5.1	0
Numbers of white blood cell(10^9^ L^−1^)	7.14 ± 3.67	7.09 ± 3.56	7.20 ± 2.98	7.98 ± 3.52
Numbers of PBMC(10^9^ L^−1^)	0.44 ± 0.25	0.43 ± 0.26	0.44 ± 0.24	0.48 ± 0.21
Numbers of CD8(+)(10^9^ L^−1^)	0.26 ± 0.14	0.23 ± 0.12	0.25 ± 0.15	0.24 ± 0.11
Percentage ratio of CD28(+)/CD28(-)	43.4 ± 22.1/56.6 ± 33.5	49.1 ± 26.5/57.1 ± 37.8	46.9 ± 22.1/53.1 ± 39.0	55.1 ± 29.2/42.6 ± 29.3
C-reactive Protein(mg L^−1^)	6.39 ± 2.86	6.26 ± 3.02	6.41 ± 3.27	5.22 ± 1.45

^a^Number of chronic disease (multimorbidity) was calculated using 10 age-related/chronic diseases including sustained/primary hypertension, primary hyperlipidemia, type 2 diabetes, coronary atherosclerotic heart disease, chronic atrial fibrillation, preclinical valvular disease, preclinical chronic obstructive pulmonary disease, subclinical chronic liver disease, subclinical chronic renal disease, subclinical thyroid disease and long-term(subclinical > 5 years) tumor survivor

^b^Number of daily medications were calculated by the number of drugs necessary to treat (NNTs). It was calculatedusing15 categories of pharmaceutical administration,including:5 oral antihypertensive drugs (angiotensin receptor blockers, angiotensin-converting enzyme inhibitors, calcium channel blockers, beta-receptor blockers, and hydrochlorothiazide); 4 oral hypoglycemic drugs (sulfonylureas, metformin, glucosidase inhibitors, and thiazolidinediones); insulin or insulin analogues; 2 lipid lowering drugs(statin and fibrate); aspirin; clopidogrel; trimetazidine; CoenzymeQ10; and isosorbidemononitrate sustained-release capsules

### General profiles of circRNA microarray

Overall, 2171 circRNAs were significantly up-regulated among the four comparison groups (N_C1(Q1vsQ2)_ = 478, N_c2(Q3vsQ4)_ = 272,N_C3(Q1vsQ3)_ = 671,N_C4(Q2vsQ4)_ = 741). (*For the concise aim, we abbreviated circular RNA as “circRNA” in the following manuscript).* In these same groups, 1563 circRNAs were significantly down-regulated (N_C1(Q1vsQ2)_ = 337,N_c2(Q3vsQ4)_ = 332,N_C3(Q1vsQ3)_ = 438,N_C4(Q2vsQ4)_ = 456). We ranked the circRNAs according to fold-change (FC) in expression levels of the 4 comparison profiles and listed the Top10 candidates (“Top 10”) of each comparison in Additional file 1: Table S1.

### Identification of Top-5 miRNAs according to mirSVR

For each circRNA with fold changes in expression levels greater than 2 in the four comparison groups, we ranked their targeted miRNAs according to mirSVR scores and identified the 5 highest ranking candidates (“Top 5”) further analysis (for details see Additional file 2: Online Supplemental Excel 1). We then utilized Cytoscape to construct circRNA-miRNA networks of interaction between those circRNA exhibiting a 2-fold or greater change in expression (up-regulated and down-regulated expression) and their Top 5 targeted miRNAs (designated as “Top-5 network”) (Additional file 3: Figure S1). Since the number of targeted miRNAs that interplayed with each circRNA in these two networks was almost equal (N_miRNA_ = 5), the profiles of these two networks appeared to be similar. There were more molecular interactions in the down-regulated Top-5network (2338 nodes with 7723 edges) than in the up-regulated Top-5 network (1865 nodes with 5558 edges).

### Identification of overlapping circRNAs between C1 and C4

Until now, the molecular interactions between most circRNAs and their target miRNAs have been addressed by theoretical prediction based on the principle of base-paring. Moreover, for any one circRNA, at least more than ten miRNAs have been predicted as potential targets. Therefore, it can be challenging to define which circRNA molecules play more critical or pivotal roles in a complex circRNA-miRNA network. Here, we supposed that circRNAs that interact with more miRNA targets play a more significant role in the general circRNA-miRNA network, than do those circRNAs that interact with fewer targets. Based on this hypothesis, we designed a step-wise strategy to optimize potential candidate circRNAs.

Firstly, among the up-regulated and down-regulated Top-5 networks, the Top-10 % miRNAs ranked by miRNA-dependent degree (D) were optimized for next step. In the re-ranked Top-10 % up-regulated network, 75 up-regulated miRNAs (Additional file 1: Table S3-1) with degree values ranging from 22 to 60 were selected. The highest degree of miRNA was hsa-miR-136-5p (D = 60), indicating that it interplayed with 60 different circRNAs in this network. In the re-ranked Top-10 % down-regulated network, 80 miRNAs with degree value ranging from 28 to 81 were selected (Additional file 1: Table S3-2). The miRNA hsa-miR-608 exhibited the highest degree (D = 81), indicating that it was regulated by 81 different circRNAs in this network. The distribution of these Top-10 % miRNAs is depicted in Additional file 3: Figure S2. Using these Top-10 % miRNAs, up-regulated and down-regulated miRNA-circRNA networks (designated as Top-10 % network-mi) were re-enriched on the basis of the fundamental miRNA-circRNA connectivity (Additional file 3: Figure S3). The distribution of degree of the miRNAs of the re-enriched Top-10 %-mi network is depicted in Additional file 3: Figure S4.

Then, we re-ranked the Top-10 % network according to circRNA-mediated degree (designated as Top10%-network-ci). In this re-ranked data set, the degree of up-regulated circRNAs ranged from 1 to 12. The circRNA100550 exhibited the largest degree (D = 12), indicating that it interplayed with 12 miRNAs. The degree of down-regulated miRNAs ranged from 1 to 15. The circRNA100988 exhibited the largest degree (D = 15), indicating that it interacted with 15 miRNAs. The distribution of the degree of the circRNAs of the Top-10 %-ci network is depicted in Additional file 3: Figure S5. From the figure, we could observe that a majority of the circRNAs interacted with only one or two miRNAs (237 circRNAs of D = 1 and 307 circRNAs of D = 2). In order to optimize intracellular validation analyses, we selected a subset of circRNAs of the largest Degree as the candidates. Here, we optimized our parameters to select circRNAs with degree values greater than 9 (Top Degrees, D ≥ 9) and enrolled the 12 up-regulated circRNAs and the12 down-regulated circRNAs with the highest degree values for intracellular validation studies.

### Venn analysis for overlapped circRNA identification by crosstalk analysis

To determine which circRNAs exhibited overlapping expression between the C1 and C4 comparison groups, Venn analyses were performed using the selected 12 up-regulated and 12 down-regulated circRNAs (The Venn’s diagram is depicted in Additional file 3: Figure S6). The details of between-group comparisons are also shown in Table 1. Four of twelve up-regulated circRNAs were expressed in both the C1 and C4 groups. Therefore, we employed qPCR to validate the intracellular expression of these four circRNAs in their respective T cell subsets of origin. Unexpectedly, none of the 12 candidate circRNAs mediated crosstalk in the down-regulated network. To compare the same number of circRNAs/molecules as in the up-regulated network, we selected four circRNAs (N = 4, Top-2 Fold Changes in C1 or C4, respectively) for the intracellular validation in the down-regulated network.

### Cellular validation using qPCR

**Fig. 1:**
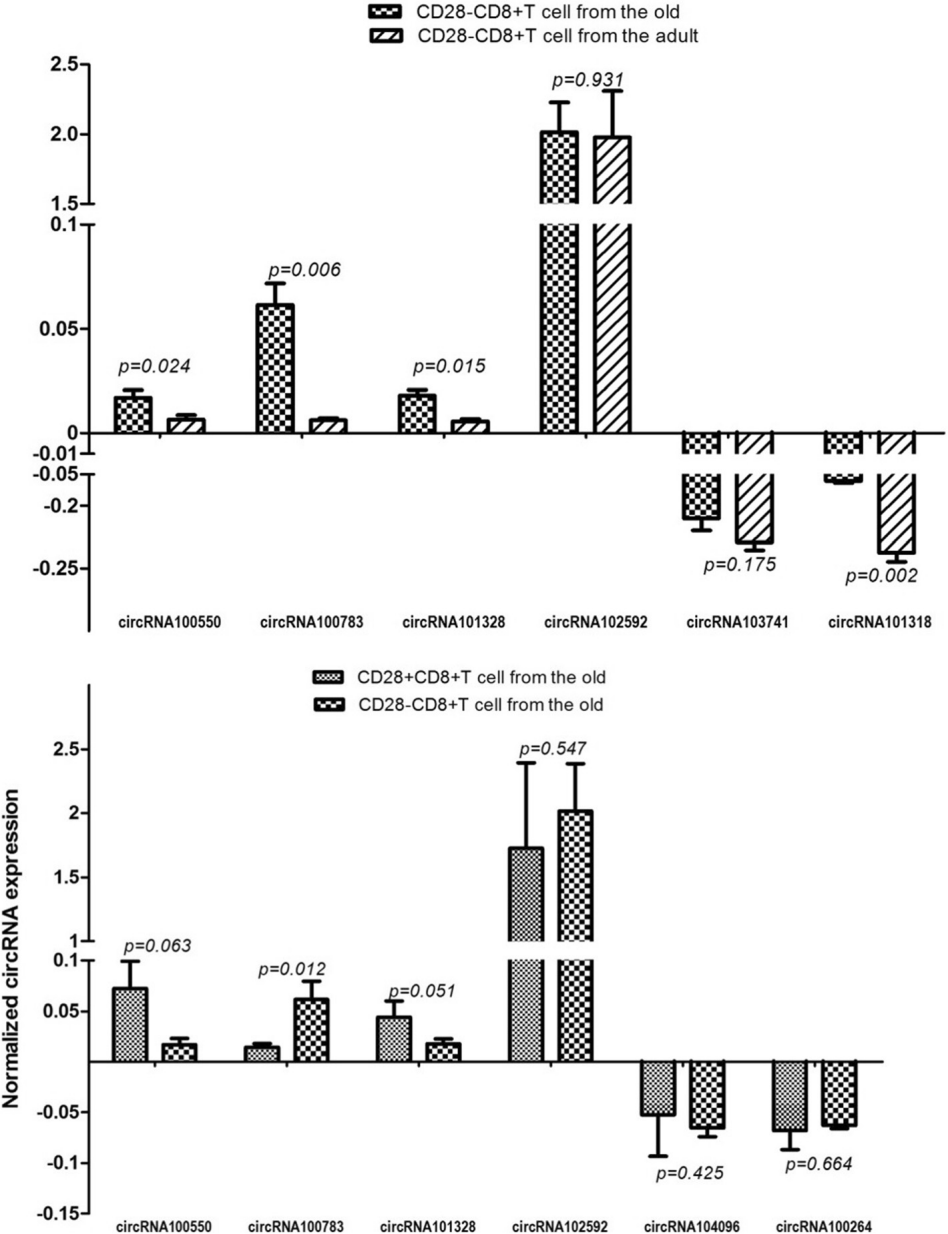
The intracellular validation of candidate circRNAs in C1 and C4. Validation of intracellular circRNA was performed using quantitative polymerase chain reaction (qPCR; in triplicate) in three randomly selected RNA samples. The level of intracellular expression of the validated circRNA was the average of these three samples. Prior to determination of the average, the normalized intracellular expression was calculated by the ratio of intracellular expression to microarray expression. Four up-regulated circRNAs (circRNA100550, circRNA100783, circRNA101328 and circRNA102592) and two down-regulated circRNAs with Top-2 Degree (circRNA103741 and circRNA101318) were validated in C1, respectively (**a**). Simultaneously, the same four up-regulated circRNAs and another two down-regulated circRNAs with Top-2 degree (circRNA104096 and circRNA100264) were validated in C4, respectively (**b**). Shown from the figure, only circRNA100783 is significantly differentially-expressed in both C1 and C4. Therefore, we supposed circRNA 1000783 might be a potential biomarker of immunosenescence

The relative intracellular expression (ratio of intracellular expression to microarray expression) of the aforementioned 8 circRNAs is shown in Fig. 1. At the intracellular level, we found that only circRNA100783 expression significantly differed between the C1and C4 groups. This indicated that the expression of circRNA100783 was affected by time (C4) and antigen exposure (C1) during CD28-related CD8(+)T cell ageing. Therefore, we propose that this circRNA100783 is a potential biomarker in ageing T lymphocytes.

### circRNA100783-targeted miRNA-mRNA network prediction and annotation

**Fig. 2:**
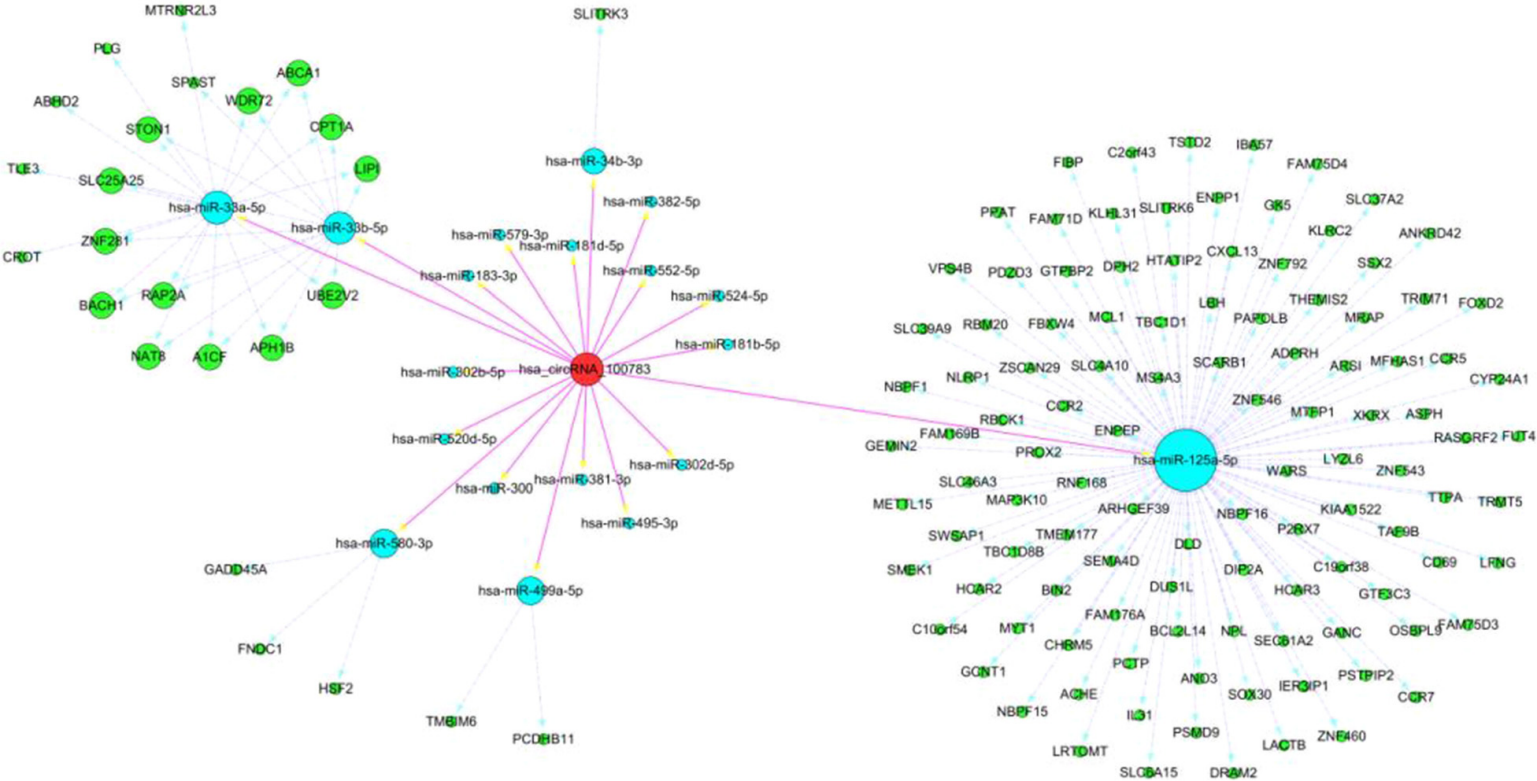
The biomathematical predicted circ000783-targeted circRNA-miRNA-mRNA/gene network. The circ000783-targeted circRNA-miRNA-mRNA/gene network is predicted based on sequence-pairing prediction. There are 73 miRNAs and 1930 genes being targeted in the present circ000783--miRNA-mRNA/gene network (miRNA-dependent cutoff value -0.11, mRNA-dependent cutoff value -0.38). Shown in this figure, miR-125a-5p exhibited the highest degree, followed by miR-33a-5p,miR-33b-5p,miR-580-3p,miR-499a-5p and miR-34b-3p

According to the initial array data analysis, the genomic locus of circRNA100783 is on chromosome 11and the predicted sequence of the best linear transcript isuc001mul.1. The molecular interaction of circRNA100783 with its Top-5 miRNA targets is depicted in Additional file 1: Table S4. Supposing that circRNA100783 is an upstream molecular sponge in its circRNA-miRNA-mRNA network, we predicted a circRNA100783-miRNA-gene network using Targetscan and miRanda. A total of Top-18 miRNAs (miRNA-dependent cutoff value -0.11) andTop-6 miRNAs (mRNA-dependent cutoff value -0.38) were finally determined in this network (Fig. 2). Using DAVID function annotation, circRNA100783 was predicted to be associated most strongly with alternative splicing, as well as with splice variation and phosphoprotein function (Additional file 3: Figure S7). Given the strong correlation of the biogenesis of circRNAs with splicing and splice variation, it is possible that circRNA100783 functions in the regulation of a phosphoprotein that is involved in a molecular mechanism that leads to the age-related phenotypic loss of CD28 from CD8(+) T cells.

In the present study, we employed high-throughput circRNA microarrays to construct profiles of differentially expressed circRNAs in CD28-associated CD8(+)T cells in the elderly and adult subjects (C1,C2,C3 and C4). Then we used a step-wise bioinformatics protocol to optimize the Top differentially expressed candidate circRNAs, and then identify those circRNAs having expression profiles that overlap between the CD28(+)CD8(+)T cells and CD28(-)CD8(+)T cells from elderly donors (C1), as well as between the CD28(-)CD8(+)T cells from elderly and adult controls (C4). To narrow the range of the candidates we employed a step-wise optimizing strategy that consisted of ranking of RNAs (circRNAs or miRNAs) by degree values, based on their circRNA-miRNA molecular connectivity. Finally, the commonly expressed candidate circRNAs in C1 and C4 were validated in their respective T cell populations. Among these, the up-regulated circRNA100783 was the only candidate molecule that appeared to play both antigen-dependent (C1) and age-dependent (C4) roles in CD8(+) T cells during ageing. Therefore, circRNA100783 may serve as a novel biomarker of immunosenescence.

Several sources of heterogeneity could hamper ageing studies and we designed our analysis to minimize this pitfall. First, “absolute” healthy seniors were rare, while the common elderly population was affected with numerous chronic diseases (multimorbidity). Moreover, the increased heterogeneity of clinical phenotypes in the elderly was also correlated to age. To minimize this clinical heterogeneity, we enrolled representative elderly patients characterized as having “continuously asymptomatic manifestation” or “apparent health”, the phenomena of which are rather prevalent in the general population. Second, over a lifetime, individuals may be exposed to several types of antigens that contribute to T cell immunosenescence. One characteristic phenotype is the eventual loss of CD28 from the CD8(+) T cell surface (CD28 exhaustion). Cytomegalovirus (CMV) antigens are believed to elicit the greatest clonal expansion of CD8(+) T cells in healthy individuals during ageing [6, 7, 22]. Moreover, infection by CMV results in changes in gene expression in the aged immune system that are not observed in CMV-negative individuals [23]. Thus, to diminish the between-antigen heterogeneity, we limited our studies to subjects who tested seropositive for CMV according to microarray detection. Third, we examined samples of CD28(+)CD8(+) T cells and CD28(-)CD8(+) T cells from the same individuals, as well as among the elderly and controls groups.

A large amount of RNA is required to perform circRNA microarray analysis. However, even after increasing the blood sample size to 50 mL, we could not isolate a sufficient amount of RNA from CD28(+)/(-)CD8(+) T cell subsets collected from elderly subjects. We propose that either the number of CD8(+)T cells or the total RNA from each cell, or both, decreased during ageing. Typically, cellular circRNAs are in relatively low abundance compared to their linear counterparts [24] and, therefore, we found it necessary to pool total RNA from several donors into one sample. To avoid potential molecular interaction, we constructed age-related subgroups of old donors, instead of using a random pooling protocol, to decrease the between-individual heterogeneity in the elderly group. Consequently, the elderly group was partitioned into three age-dependent subgroups: the adult-old subgroup (aged 65-74, n = 8); the middle-old subgroup (aged 75-84, n = 7) and the old-old subgroup (aged 85-94, n = 6).

Although 7,112 human circRNAs have been identified, and are estimated to comprise at least 10 % of the transcripts accumulating from their loci, most circRNAs are expressed in only a few cell types and in low abundance [25]. Based on the current literature, it is unclear if, and to what extent, circRNAs regulate target miRNAs through sequence pairing. The elucidation of the molecular mechanism of circRNA-miRNA interactions would require numerous and extensive binding studies. A previous report showed that low levels of circRNAs may not be sufficient to affect the target molecules (Denzler et al. 2014). In view of this, we supposed that most circRNAs in the integrated circRNA-miRNA network played weak and transient interactions. Therefore, we designed a step-wise biomathematical protocol to identify candidates among differentially-expressed circRNAs based on their connectivity to target miRNA (independent of fold change). We considered that circRNAs which exhibited greater connectivity to miRNAs than did other circRNAs were more likely to play a role in the CD28 loss from CD8(+) T cell surfaces during ageing. After a basic circRNA-miRNA connectivity network was constructed, a step-by-step biomathematical analysis was performed.

The strategy we developed to analyze the expression of candidate circRNA consisted of three steps: 1) ranking theTop-5 miRNA targets in the circRNA-miRNA network and selecting the Top-10 % miRNAs according to miRNA-mediated connectivity to construct the Top-10 %-mi network, 2) re-ranking the Top-10 %-mi network according to circRNA-mediated connectivity to obtain a Top-10 %-ci network and, 3) identifying candidate circRNAs with a degree value of 9 or greater in the Top-10 %-ci network. Using the step-wise analysis, twelve up-regulated circRNAs and down-regulated circRNAs were identified as candidates for intracellular validation. We then employed a Venn diagram to explore which of these circRNA molecules exhibited overlapping expression in both the C1 and C4 comparison groups (Additional file 3: Figure S6). This permitted us to visualize changes in expression due to antigen-accumulated alterations (C1) and age (4). As shown in Table 1 (upper panel), a total of twelve up-regulated candidate circRNAs were distributed in the four comparison groups. Of the six up-regulated circRNAs from C1 and ten up-regulated circRNAs from C4, four molecules (circRNA100550, circRNA100783, circRNA101328 and circRNA102592) exhibited overlapping expression in the two groups. We subsequently validated these circRNAs in the corresponding T cell subsets using qPCR. However, none of the down-regulated circRNAs were expressed in the four comparison groups (Table 2, lower panel). Therefore, we selected for validation two circRNAs which ranked in the Top-2, according to Fold Change, in the two comparison groups. Among these four up-regulated overlapping circRNAs, only the differential expression of circRNA101318 was validated by intracellular qPCR detection.

**Table 2:** The microarray Fold Change of the candidate circRNAs that were step-wise selected

circRNA	Degree	C1:Q1 vs Q2	C4:Q2 vs Q4	*C2:Q3 vs Q4*	*C3:Q1 vs Q3*
Up-regulated circRNAs		Fold Change			
hsa_circRNA_100550^a^	12	2.1051912	2.7916539	*4.9934264*	
hsa_circRNA_001259	10	2.9541118			*4.9224802*
hsa_circRNA_101635	10		2.6649119		*2.339966*
hsa_circRNA_102592	10		2.1983682		*2.1898953*
hsa_circRNA_100315	10		3.8537081	*2.9419454*	
hsa_circRNA_101358	9		5.5122991	*2.8866116*	*3.6304114*
hsa_circRNA_101384	9		10.8204701	*2.7116116*	*3.3926842*
hsa_circRNA_102592^a^	9	3.8876708	2.7185627		*6.3889501*
hsa_circRNA_400075	9		6.9227289	*5.663918*	*4.0526795*
hsa_circRNA_104981	9	4.8466812			
hsa_circRNA_100783^a^	9	3.1348047	4.1833976	*7.213918*	
hsa_circRNA_101328^a^	9	2.2217774	2.2872726		*2.6027261*
Down-regulated circRNAs		Fold Change			
hsa_circRNA_100988	15		5.6605839	*2.086644*	*2.938461*
hsa_circRNA_100989	12			*5.4144951*	*2.6900132*
hsa_circRNA_103801	10		2.1387435	*3.3525614*	
hsa_circRNA_104646	10	2.5271826		*2.1783708*	
hsa_circRNA_100807	10		2.4750588	*3.7937131*	
hsa_circRNA_101318^c^	10		7.3547169	*6.3808594*	*3.0753515*
hsa_circRNA_100264^b^	10	4.3266365			*3.2787265*
hsa_circRNA_103741^c^	10		7.7727788	*2.5595041*	*3.8058778*
hsa_circRNA_103572	10	3.2713424			
hsa_circRNA_104096^b^	10	3.2940966			*2.0907926*
hsa_circRNA_104266	9		4.5747819	*2.0283626*	*2.3366408*
hsa_circRNA_103623	9	2.1655964			*2.2533911*

^a^Up-regulated overlapping circRNAs of C1 and C4 (FC underlined)

^b^Down-regulated circRNAs with Top-2 FC ofC1

^**c**^Down-regulated circRNAs with Top-2 FC of C4

Note: C1:Q1 vs Q2 (CD28(+)CD8(+)T cells vs CD28(-)CD8(+)T cells in the elderly); C2:Q3 vs Q4 (CD28(+)CD8(+)T cells vs CD28(-)CD8(+)T cells in the adult); C3 = Q1 vs Q3 (CD28(+)CD8(+)T cells in the elderly vs the adult); C4 = Q2vsQ4 (CD28(-)CD8(+)T cells in the elderly vs the adult)

Until now, only two circRNAs have been functionally verified as miRNA sponges among thousands of detected circRNAs with predicted miRNA binding sites [16, 17]. However, studies of SNP density at miRNA seed sites in 3' untranslated regions and flanking sequences, as well as at random sites, suggest that many of the predicted miRNA binding sites in circRNAs are functional and under similar selective pressure as are miRNA binding sites in mRNAs [26]. In the present study, we also predicted that circRNA100783 may function as a “sponge” to its targeted miRNAs in the predicted circRNA-miRNA-mRNA network. Then we used DAVID bioinformatics resources (Huang da et al., 2009) and KEGG pathway analysis (Kanehisa et al., 2006) to functionally annotate the predicted target genes of circRNA100783. According to our annotation, circRNA100783 is predicted to be involved in three possible functions including alternative splicing, splice variation and association with phosphoprotein(s) (Additional file 3: Figure S7).

## Conclusions

In the present study, we used the cross-comparison of circRNA microarrays and step-wised bioinformatics to determine that circRNA100783 may be involved in the loss of CD28 in CD8(+)T cells during ageing. A circRNA-miRNA-gene network suggests that circRNA100783, might regulate phosphoprotein-related signal transduction on CD28-dependent CD8(+)T cell ageing. A longitudinal study may verify circRNA100783 as a potential intracellular biomarker of immunosenescence.

## Methods

### Participants

The elderly donors were chosen from a group of participants in the Longitudinal Project on Witnessing Ageing (LPWA) in the Geriatrics Department of the First Affiliated Hospital of Harbin Medical University [27]. Each individual’s annual medical documentation, including chronic disease, long-term clinical complaints, physical examinations, laboratory measurements, imageological diagnoses, daily life style, daily necessary medication, and pharmaceutical adjustments were comprehensively reviewed. Subjects aged 65 to 94 who were continuously asymptomatic in the recent twelve months were initially enrolled as “apparently healthy elderly individuals”. After testing for latent human cytomegalovirus (CMV) infection by CMV-IgG, the CMV- seronegative subjects were excluded from the study. Adult CMV-seropositive healthy donors aged 25-35, who volunteered in the Geriatric Department, were enrolled and assessed by medical examination. All participants agreed to participate in the study with written content. The study was approved by the First Affiliated Hospital of Harbin Medical University of Medicine Ethic Committee and the research was carried out in compliance with the Helsinki Declaration.

### Isolation of CD28(+)CD8(+) T cells and CD28(-)CD8(+)T cells by flow cytometry sorting

A 20 mL intravenous blood sample was taken from each participant in the morning. Peripheral blood mononuclear cells (PBMCs) derived using Ficoll-Hypaque Plus gradient centrifugation were incubated in fluorescence-activated cell sorter (FACS) buffer with anti-CD8-phycoerythrin (PE; BD Biosciences) and anti-CD28-fluorescein isothiocyanate (FITC; BD Biosciences) for 30 min. Then PBMCs were washed in FACS buffer, resuspended in 1 % paraformaldehyde in FACS buffer, and sorted in a FACSCanto flow cytometer (BD Biosciences) in the National Key Laboratory of Harbin Vegetarian Research Institute. CD28(-)CD8(+)T cells and CD28(+)CD8(+)T cells from each donor were collected using two sterile collection tubes, respectively, for RNA extraction and analyses.

### circRNA microarray hybridization

Total RNA from either CD28(-)CD8(+)T cells or CD28(+)CD8(+)T cells from the same subject was extracted and quantified using the NanoDrop ND-1000. RNA was prepared and stored at -80 °C for the cellular validation. RNA from each sample was subjected to microarray analysis and hybridization according to the manufacturer’s protocol (Arraystar). An aliquot consisting of 10 % of the RNA from each sample was reserved for qRNA analysis.

### circRNA microarray data analysis

**Fig. 3:**
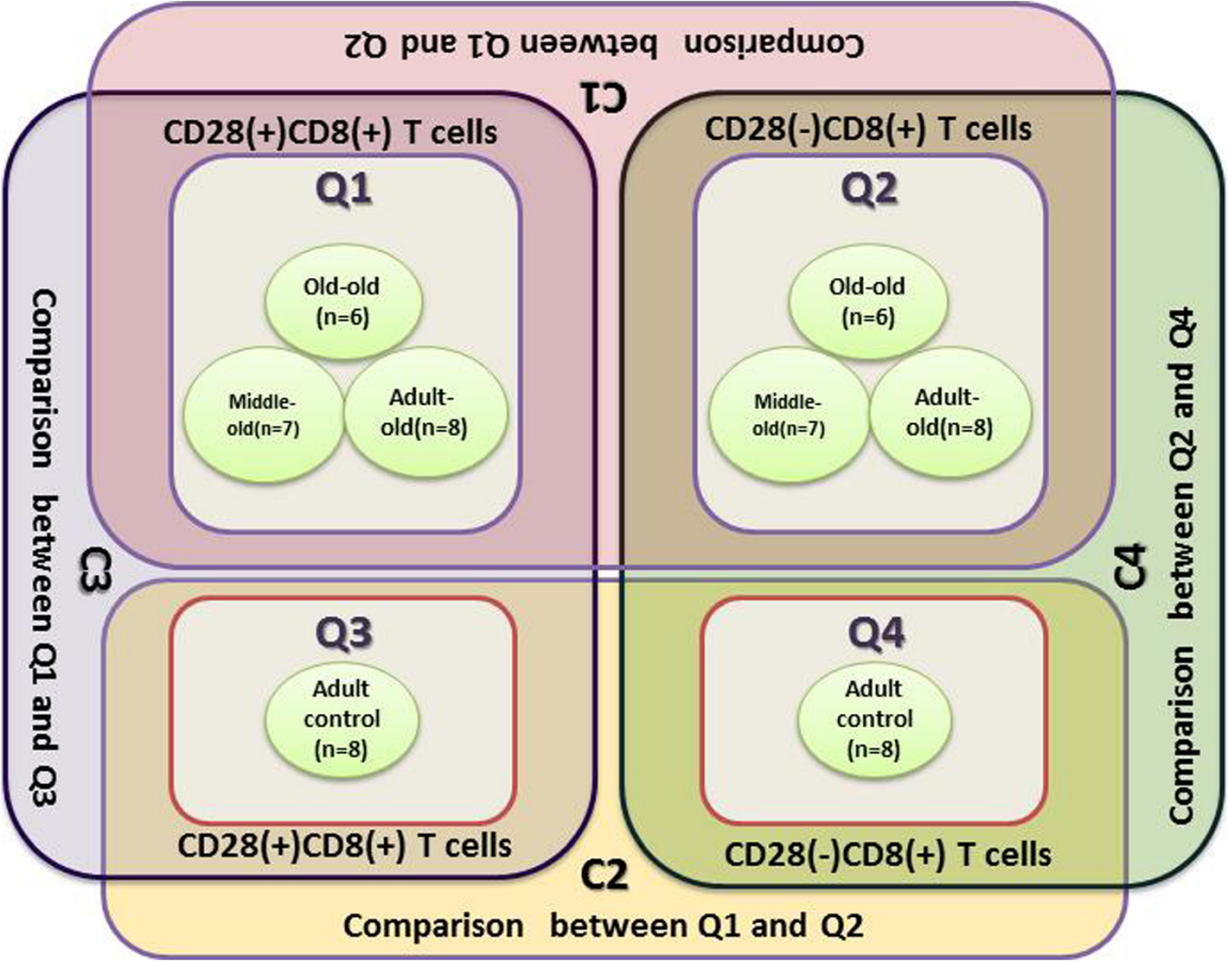
Schematic strategy of pooling circRNA microarray profiles. Eight microarrays are depicted by 8 green circles. After eight microarray profiles were individually acquired, three microarray profiles detecting CD28(+)CD8(+)T cells from three age-dependent elderly subgroups were pooled as Q1,and three microarray profiles detecting CD28(-)CD8(+)T cells from three age-dependent elderly subgroups were pooled as Q2. Consequently, four cross-group comparisons were performed as: Comparison1 (*C1:Q1 vs Q2* indicating CD28(+)CD8(+) *vs* CD28(-)CD8(+) T cells in the elderly); Comparison2 (*C2: Q3 vs Q4* indicating CD28(+)CD8(+) *vs* CD28(-)CD8(+)T cells in the adult); Comparison 3 (*C3:Q1 vs Q3* indicating CD28(+)CD8(+)T cells in the elderly *vs* in the adult); and Comparison4 (*C4: Q2 vs Q4* indicating CD28(-)CD8(+)T cells in the elderly *vs* in the adult)

Eight RNA samples extracted from either CD28(+)CD8(+) T cells or CD28(-)CD8(+) T cells were pooled into one RNA sample for one microarray detection (i.e. eight microarrays were performed). Then, data from 6 microarrays using RNA isolated from the elderly group were pooled into groups Q1 (CD28(+)CD8(+)) or Q2 (CD28(-)CD8(+)), respectively. Data from-two microarrays using RNA isolated from the adult cohort were analyzed as Q3 (CD28(+)CD8(+)) or Q4 (CD28(-)CD8(+)), correspondingly. Figure 3 schematically depicts the grouping of the eight circRNA microarrays and the four cross-group comparisons (C1 = Q1 vs Q2; C2 = Q3 vs Q4; C3 = Q1vs Q3; C4 = Q2 vs Q4) that were performed (see Additional file 1: Table S2 for the pooling and grouping protocol).

### Identification of the circRNA-miRNA connectivity

Using microarray analysis we initially identified the circRNAs that exhibited a more than 2-fold change in expression. Then their respective miRNA targets, which were predicted based on sequence-pairing, were identified by TargetScan and verified by miRanda; thus the basic circRNA-miRNA connectivity was established. All subsequent biomathematical analyses were based on this basic molecular connectivity.

### Step-wise biomathematical circRNA candidate optimization

After the basic circRNA-miRNA connectivity was determined, we focused our efforts on finding overlapping circRNA profiles between the C1 and C4 comparison groups. However, among the Top-10 circRNAs listed in Additional file 1: Table S1, none were commonly expressed in C1 and C4. Even when we expanded our list of candidates to include the Top-50 circRNAs, we observed no overlapping circRNA expression in C1 and C4. Therefore, we employed a step-wise biomathematical analysis to identify potential overlapping circRNAs (Animated details are depicted in Additional file 4: Online Supplemental PPT1).

First, to focus the targeted miRNA profile, the miRNA support vector regression (mirSVR) algorithm was used to score and rank the efficiency of the predicted microRNA targets (Betel et al. 2010). Accordingly, for each circRNA exhibiting greater than a 2-fold expression level, we identified 5miRNAs with the highest mirSVR (Top-5). Thus a “Top-5-mi” circRNA-miRNA network was established (one circRNA connecting to five miRNAs, miRNA-mediated Degree = 5). Second, this Top-5 circRNA-miRNA network was re-ranked with respect to circRNA-mediated connectivity (one miRNA connecting to N circRNAs, circRNA-mediated Degree = N). Then, circRNAs were ranked according to degree and the Top-10 % candidates were identified for these restricted profiles. The cutoff of 10 % was selected empirically according to our previous bioinformatics experience. Using these Top-10 % circRNAs, a “re-enriched” Top-10 % miRNA-circRNA network was established (“Top-10 %-mi”). Third, this Top-10 % network was re-ranked according to the miRNA-mediated degree (one circRNA connecting M miRNAs,Degree = M). Our final investigation focused on circRNAs that exhibited degrees of 9 or greater in this re-ranked Top-10 % network (“Top-10 %-ci”). The selectedTop-9 circRNAs were subjected to Venny analysis to discern overlapping candidates (Table 1).

### Validation of candidate circRNAs using qRT-PCR

Validation of circRNA was performed using quantitative polymerase chain reaction (qPCR; in triplicate) in three randomly selected RNA samples. Divergent primers (instead of commonly used convergent primers) were designed and optimized for eight circRNAs. The sequence of circRNA results were acquired from the database “circBase”. The Glyceraldehyde 3-phosphate dehydrogenase (GAPDH) housekeeping gene was used as a control (Details are shown in Additional file 1: Table S5). To ensure the accuracy of the results, we verified the target specificity of the PCR primers using BLAST (http://blast.ncbi.nlm.nih.gov/Blast.cgi). The appearance of a single-peak in the melting curve indicated the specificity of the PCR results. The data were analyzed using the ΔCt method. Validation was designated by the ratio of the differential cellular expression/differential microarray expression.

### Annotation and function prediction for the validated circRNAs

Validated candidate circRNAs were used as seeds to enrich a circRNA-miRNA-gene network according to the cutoff value determined using mirSVR. The predicted gene functions in the networks were annotated using Gene Ontology (GO) and KEGG Pathway using Database for Annotation, Visualization and Integrated Discovery (DAVID) [28].

### Statistical analysis

Quantile normalization and subsequent data processing were performed using the R software package. All other statistical data were analyzed and visualized by GraphPad Prism5.0 (GraphPad Software, La Jolla, CA). The significance of qPCR validation of C1 and C4 were tested by Student’s *t* test (*P* < 0.05)and was considered statistically significant.

## Additional files

Additional file 1: Table S1. The top 10 differentiated circRNAs ranking by Fold Change (FC) in four comparison groups. **Table S2.** The RNA pooling and grouping protocol of the eight microarrays. **Table S3-1.** Distribution of the Top 10% Degree of up-regulated miRNAs (Degree range=22-60, n=75. **Table S3-2.** Distribution of the Top 10% Degree of down-regulated miRNAs (Degree range=28-81, n=80. **Table S4.** Five detailed annotation for the circRNA/miRNA interaction (circ100783 and its Top-5 predicted miRNA targets). **Table S5.** The detailed characteristicsof qRT-PCR for eight candidate circRNAs. (DOCX 344 kb) Additional file 2: The original microarray results could be seen in the Online Supplemental Excel. (XLS 19149 kb) Additional file 3: Figure S1. Top-5 circRNA-miRNA networks using miRNAs with the 5 highest degrees. **Figure S2.** Distributions of miRNAs of Top10% Degrees in the Top-5 networks. **Figure S3.** Re-enriched networks using up-regulated miRNAs (left) and down-regulated miRNAs(right) of Top-10% highest Degree in the original Top-5 network. **Figure S4.** Distributions of Degree of the miRNAs in the re-enriched Top-10%-mi network. **Figure S5**. Distribution of Degree of the circRNAs in the re-enriched Top10%-ci network. **Figure S6A.** Venn’s diagram for optimized circRNA candidates between four groups. **Figure S7.** The screenshot of the online annotation for the targeted genes in the circRNA100783-targeted miRNA-gene network determined by DAVID annotation. (DOCX 1500 kb) Additional file 4:
**Illustration of** **step-wised** **optimization on overlapping circRNAs between C1 and C4.** (PPTX 66 kb)
