# Supplementary material for: Comprehensive circular RNA profiling reveals that circular RNA100783 is involved in chronic CD28-associated CD8(+)T cell ageing
Source: Immun Ageing. 2015 Oct 8;12:17. doi: 10.1186/s12979-015-0042-z (PMC4597608; doi:10.1186/s12979-015-0042-z)
Supplement: Additional file 1: Table S1. — The top 10 differentiated circRNAs ranking by Fold Change (FC) in four comparison groups. Table S2. The RNA pooling and grouping protocol of the eight microarrays. Table S3-1. Distribution of the Top 10% Degree of up-regulated miRNAs (Degree range=22-60, n=75. Table S3-2. Distribution of the Top 10% Degree of down-regulated miRNAs (Degree range=28-81, n=80. Table S4. Five detailed annotation for the circRNA/miRNA interaction (circ100783 and its Top-5 predicted miRNA targets). Table S5. The detailed characteristicsof qRT-PCR for eight candidate circRNAs. (DOCX 344 kb) [file 12979_2015_42_MOESM1_ESM.docx]

**Additional file 1**

Table 1S. The top 10 differentiated circRNAs ranking by Fold Change (FC) in four comparison groups

| Rank | | C1:Q1vsQ2 | | | C2:Q3vsQ4 | | | | C3:Q1vsQ3 | | | | C4:Q2vsQ4 | | |
| --- | --- | --- | --- | --- | --- | --- | --- | --- | --- | --- | --- | --- | --- | --- | --- |
|  |  | ID (Alias) | | FC | ID (Alias) | | FC | | ID (Alias) | | | FC | ID (Alias) | | FC |
| Up-regulated Differentiation | | | | | | | | | | | | | | | |
| 1 | | circ0001907 | | 19.4 | circ0005418 | | 38.8 | | circ0072567 | | | 12.0 | circ0005154 | | 17.3 |
| 2 | | circ0008394 | | 15.1 | circ0058048 | | 27.4 | | circ0047303 | | | 11.9 | circ0005720 | | 16.9 |
| 3 | | circ0001280 | | 15.0 | circ0001333 | | 25.1 | | circ0000420 | | | 11.8 | circ0055940 | | 16.7 |
| 4 | | circ0000072 | | 14.2 | circ0008732 | | 15.9 | | circ0001013 | | | 11.5 | circ0091024 | | 15.7 |
| 5 | | circ0000220 | | 12.3 | circ0022392 | | 15.0 | | circ0030741 | | | 11.2 | circ0078328 | | 15.1 |
| 6 | | circ0077096 | | 11.7 | circ0008460 | | 12.3 | | circ0030388 | | | 10.9 | circ0092375 | | 15.0 |
| 7 | | circ0071869 | | 11.3 | circ0091024 | | 11.4 | | circ0000828 | | | 10.4 | circ0092300 | | 14.9 |
| 8 | | circ0000835 | | 11.1 | circ0005777 | | 11.2 | | circ0092342 | | | 10.3 | circ0001414 | | 14.8 |
| 9 | | circ0072263 | | 10.5 | circ0008139 | | 11.1 | | circ0088090 | | | 10.1 | circ0000942 | | 14.5 |
| 10 | | circ0000841 | | 9.8 | circ0005838 | | 10.7 | | circ0050102 | | | 9.9 | circ0021727 | | 14.0 |
| Down-regulated Differentiation | | | | | | | | | | | | | | | |
| 1 | circ0050490 | | -15.9 | | | circ0033024 | | -14.2 | | circ0084615 | -17.2 | | | circ0009061 | -29.9 |
| 2 | circ0069660 | | -12.5 | | | circ0002807 | | -11.2 | | circ0000043 | -11.7 | | | circ0007759 | -22.4 |
| 3 | circ0037104 | | -11.4 | | | circ0073568 | | -10.9 | | circ0001411 | -11.2 | | | circ0000154 | -20.2 |
| 4 | circ0004370 | | -10.4 | | | circ0001556 | | -10.4 | | circ0007331 | -8.8 | | | circ0007108 | -19.1 |
| 5 | circ0083866 | | -9.8 | | | circ0047285 | | -7.8 | | circ0008982 | -8.5 | | | circ0061721 | -17.7 |
| 6 | circ0008258 | | -9.7 | | | circ0092363 | | -7.7 | | circ0088479 | -8.1 | | | circ0061276 | -17.5 |
| 7 | circ0029104 | | -8.9 | | | circ0021569 | | -7.7 | | circ0002105 | -7.9 | | | circ0050486 | -16.0 |
| 8 | circ0020100 | | -7.6 | | | circ0091104 | | -7.5 | | circ0008021 | -7.8 | | | circ0008902 | -15.7 |
| 9 | circ0005918 | | -7.3 | | | circ0000630 | | -7.3 | | circ0088457 | -7.4 | | | circ0000841 | -15.5 |
| 10 | circ0008982 | | -7.2 | | | circ0050486 | | -7.2 | | circ0037866 | -6.9 | | | circ0001811 | -15.1 |

C1:Q1 vsQ2 (CD28+CD8+T cells vs CD28-CD8+T cells in the elderly); C2:Q3 vs Q4 (CD28+CD8+T cells vs CD28-CD8+T cells in the young; C3=Q1 vs Q3 (CD28+CD8+T cells in the elderly vs the young); C4=Q2 vs Q4 (CD28-CD8+T cells in the elderly vs the young).

*FC=Fold Change

Table 2S: The RNA pooling and grouping protocol of the eight microarrays

| Microarray Detection in the elderly subjects (**Q1 and Q2** ) | | | |
| --- | --- | --- | --- |
| **Q1** (3 microarrays) **Q2** (3 microarrays) | | | |
| Q1-1: | pooled RNAs of CD8+CD28-T cells from 6 old-old subjects | Q2-1: | pooled RNAs of CD8+CD28+T cells from the same 6 old-old subjects |
| Q1-2 | pooled RNAs of CD8+CD28-T cells from 7 middle-old subjects | Q2-2 | pooled RNAs of CD8+CD28+T cells from the same 7 middle-old subjects |
| Q1-3 | pooled RNAs of CD8+ CD28-T cells from 8 young-old subjects | Q2-3 | pooled RNAs of CD8+ CD28+T cells from the same 8 young-old subjects |
| Microarray Detection in the adult subjects(**Q3 and Q4** ) | | | |
| **Q3** (1 microarray) **Q4** (1 microarray) | | | |
| Q3 | pooled RNAs of CD8+ CD28-T cells from 8 adult controls | Q4 | pooled RNAs from CD8+CD28+T cells of 8 adult controls |

Table 3S-1：Distribution of the Top 10% Degree of up-regulated miRNAs (Degree range=22-60, n=75）

| Degree | | Numbers | | miRNAs list |
| --- | --- | --- | --- | --- |
| 60 | 1 | | hsa-miR-136-5p | |
| 59 | 1 | | hsa-miR-103a-2-5p | |
| 50 | 1 | | hsa-miR-612 | |
| 48 | 1 | | hsa-miR-153-5p | |
| 44 | 1 | | hsa-miR-22-5p | |
| 41 | 1 | | hsa-miR-485-5p | |
| 40 | 2 | | hsa-miR-335-3p，hsa-miR-1301-3p | |
| 38 | 2 | | hsa-miR-665，hsa-miR-329-5p | |
| 37 | 1 | | hsa-miR-29b-1-5p | |
| 36 | 2 | | hsa-miR-627-3p，hsa-miR-141-5p | |
| 35 | 1 | | hsa-miR-761 | |
| 34 | 2 | | hsa-miR-608，hsa-miR-145-5p | |
| 33 | 5 | | hsa-miR-637，hsa-miR-661，hsa-miR-135b-5p，hsa-miR-30d-3p，hsa-miR-544a | |
| 32 | 3 | | hsa-miR-298,hsa-miR-452-3p,hsa-miR-135a-5p | |
| 31 | 1 | | hsa-miR-20b-3p | |
| 30 | 4 | | hsa-miR-215-3p,hsa-miR-93-3p,hsa-miR-651-3p,hsa-miR-103a-3p | |
| 29 | 7 | | hsa-miR-370-3p,hsa-miR-30b-3p,hsa-miR-670-3p,hsa-miR-660-3p,  hsa-miR-494-5p,hsa-miR-107,hsa-miR-877-3p, | |
| 28 | 4 | | hsa-miR-138-5p,hsa-miR-518c-5p,hsa-miR-449c-5p,  hsa-miR-214-3p | |
| 27 | 7 | | hsa-miR-181a-2-3p,hsa-miR-26b-3p,hsa-miR-185-3p,  hsa-miR-328-5p,hsa-miR-762,hsa-miR-152-5p,hsa-miR-338-3p | |
| 26 | 5 | | hsa-miR-766-3p,hsa-miR-330-3p,hsa-miR-330-5p,hsa-miR-619-5p,  hsa-miR-509-5p | |
| 25 | 3 | | hsa-miR-619-3p,hsa-miR-146a-3p,hsa-miR-548a-3p | |
| 24 | 3 | | hsa-miR-17-3p,hsa-miR-203a-5p,hsa-miR-765 | |
| 23 | 12 | | hsa-miR-9-5p,hsa-miR-216a-3p,hsa-miR-182-5p,hsa-miR-653-3p,  hsa-miR-520a-5p,hsa-miR-29a-5p,hsa-miR-525-5p,hsa-miR-511-5p  hsa-miR-578,hsa-miR-1264,hsa-miR-130b-5p,hsa-miR-212-5p | |
| 22 | 5 | | hsa-miR-29b-2-5p,hsa-miR-646,hsa-miR-432-5p,hsa-miR-429,  hsa-miR-891a-3p | |

Table3S-2：Distribution of the Top 10% Degree of down-regulated miRNAs (Degree range=28-81, n=80）

| Degree | Numbers | miRNAs list |
| --- | --- | --- |
| 81 | 1 | hsa-miR-608 |
| 69 | 1 | hsa-miR-103a-2-5p |
| 67 | 1 | hsa-miR-29b-1-5p |
| 61 | 1 | hsa-miR-877-3p |
| 59 | 2 | hsa-miR-138-5p,hsa-miR-1301-3p |
| 57 | 2 | hsa-miR-22-5p,hsa-miR-141-5p |
| 56 | 1 | hsa-miR-136-5p |
| 53 | 1 | hsa-miR-612 |
| 52 | 1 | hsa-miR-660-3p |
| 51 | 3 | hsa-miR-370-3p,hsa-miR-329-5p,hsa-miR-185-3p |
| 50 | 2 | hsa-miR-335-3p,hsa-miR-544a |
| 48 | 1 | hsa-miR-330-5p |
| 46 | 4 | hsa-miR-215-3p,hsa-miR-646,hsa-miR-670-3p,hsa-miR-93-3p |
| 45 | 1 | hsa-miR-485-5p |
| 44 | 1 | hsa-miR-30b-3p,hsa-miR-762 |
| 43 | 2 | hsa-miR-29a-5p,hsa-miR-511-5p |
| 42 | 1 | hsa-miR-424-5p |
| 41 | 5 | hsa-miR-637,hsa-miR-766-3p,hsa-miR-627-3p,hsa-miR-578,hsa-miR-764 |
| 40 | 4 | hsa-miR-766-5p,hsa-miR-17-3p,hsa-miR-149-3p,hsa-miR-449c-5p |
| 39 | 2 | hsa-miR-508-5p,hsa-miR-130b-5p |
| 38 | 2 | hsa-miR-661,hsa-miR-26b-3p |
| 37 | 2 | hsa-miR-761,hsa-miR-214-3p |
| 36 | 4 | hsa-miR-9-5p,hsa-miR-328-5p,hsa-miR-452-5p,hsa-miR-558 |
| 35 | 1 | hsa-miR-135b-5p |
| 34 | 4 | hsa-miR-647,hsa-miR-597-3p,hsa-miR-203a-5p,hsa-miR-153-5p |
| 33 | 3 | hsa-miR-298,hsa-miR-338-3p,hsa-miR-145-5p |
| 32 | 6 | hsa-miR-29b-2-5p,hsa-miR-518c-5p,hsa-miR-432-5p,hsa-miR-450b-5p,hsa-miR-422a,hsa-miR-19b-2-5p |
| 31 | 1 | hsa-miR-30d-3p |
| 30 | 6 | hsa-miR-765,hsa-miR-545-3p,hsa-miR-619-5p,hsa-miR-323a-5p,hsa-miR-135a-5p,hsa-miR-22-3p |
| 29 | 9 | hsa-miR-665,hsa-miR-34c-5p,hsa-miR-34b-5p,hsa-miR-889-5p,hsa-miR-15a-5p,hsa-miR-494-5p,hsa-miR-149-5p,hsa-miR-105-5p,hsa-miR-382-5p |
| 28 | 4 | hsa-miR-525-5p,hsa-miR-449b-5p,hsa-miR-452-3p,hsa-miR-197-3p |

Table 4S. Five detailed annotation for the circRNA/miRNA interaction (circ100783 and its Top-5 predicted miRNA targets)

| has-miR-338-3p VS has_circRNA_100783 |
| --- |
| 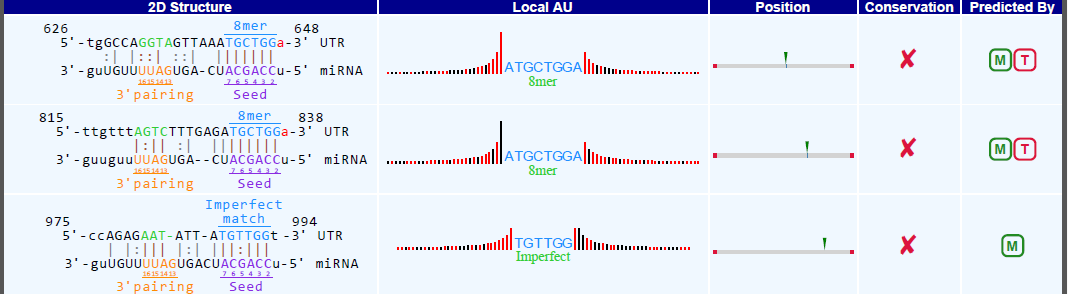 |
| hsa-miR-30d-3p VS hsa_circRNA_100783 |
| 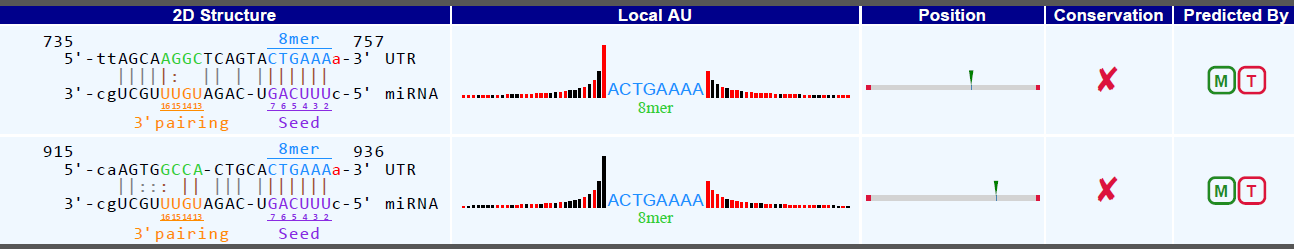 |
| hsa-miR-107 VS hsa_circRNA_100783 |
| 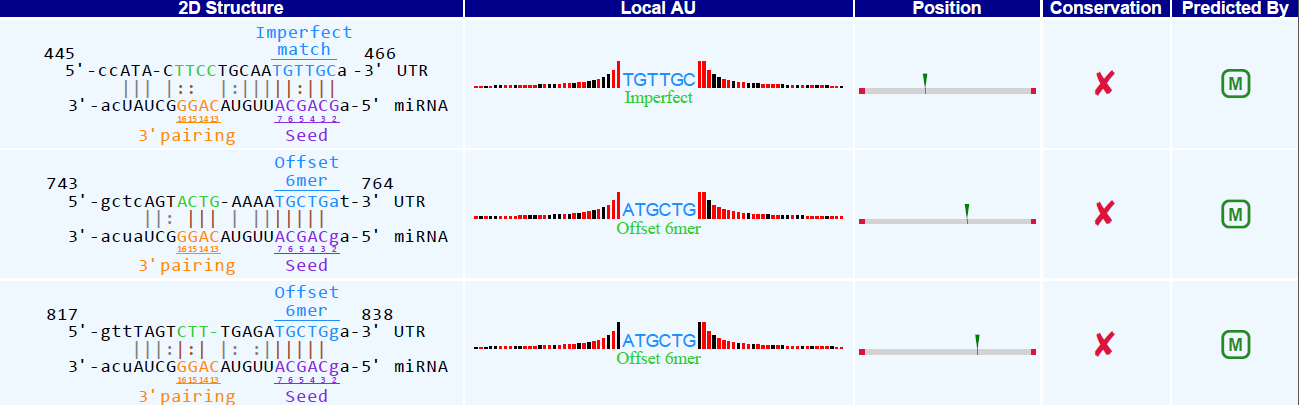 |
| hsa-miR-103a-3p VS hsa_circRNA_100783 |
| 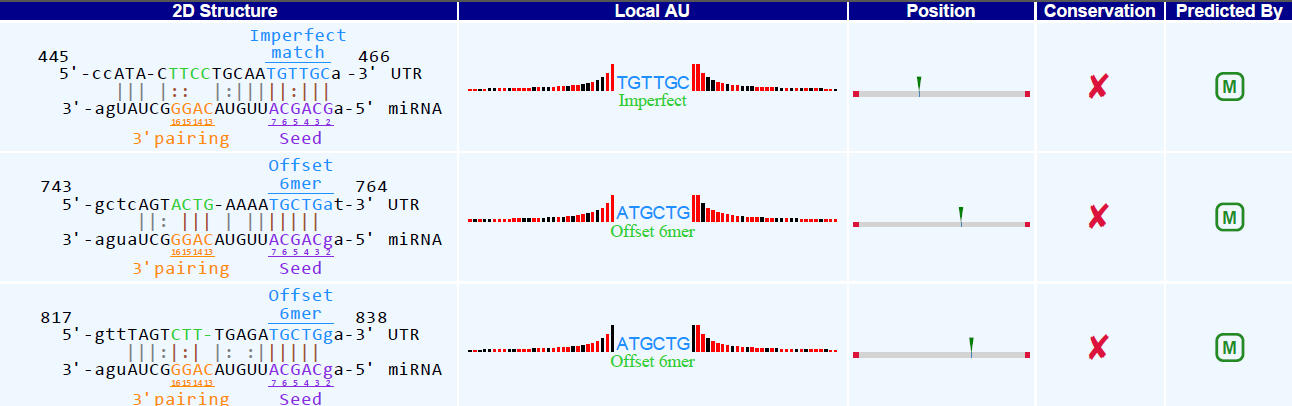 |
| hsa-miR-17-3p VS hsa_circRNA_100783 |
| 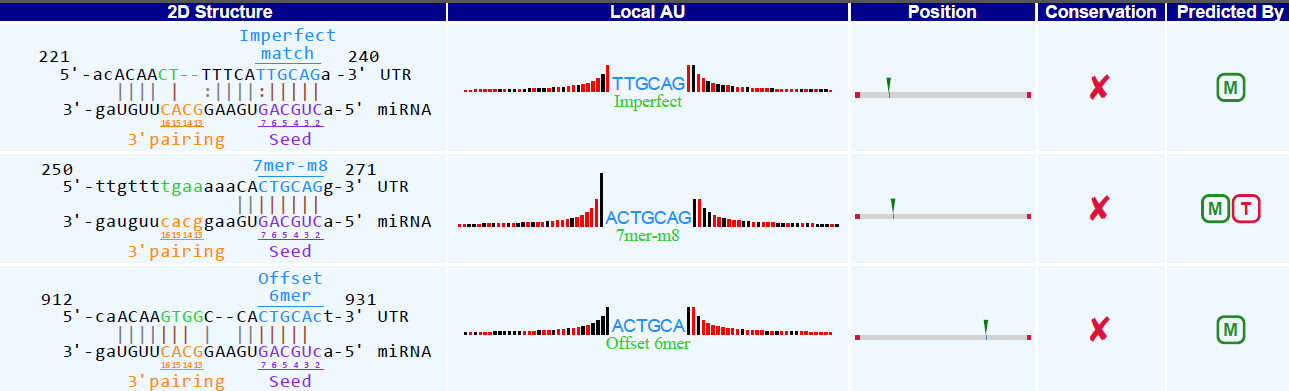 |

Table 5S.The detailed characteristicsof qRT-PCR for eight candidate circRNAs

| Target name | Primer sequence | Annealing Temperature (℃) | Product length(bp) |
| --- | --- | --- | --- |
| **Up-regulated circRNAs** | | | |
| hsa_circRNA_100550 | F:5’GCAAATCACCACCTGGAGTA3’  R:5’TTGGATTTCAGGCACATTCT3’ | 60 | 237 |
| hsa_circRNA_100783 | F:5’CCATTTTGTGAAGCCATAGAC3’  R:5’CACATAGGTCCGTGGATAGTTT3’ | 60 | 236 |
| hsa_circRNA_101328 | F:5’GGACGGCGTCACCAACCTA3’  R:5’GCACGCATTCTTTCTGGACAT3’ | 60 | 150 |
| hsa_circRNA_102592 | F:5’ACGTGTACGCAGTGACCGAG3’  R:5’GAAGCTGCTTGTACGGGTTG3’ | 60 | 94 |
| **Down-regulated circRNAs** | | | |
| hsa_circRNA_100264 | F:5’GCCAGTCAGCACATTACAGAAG 3’  R:5’TTGCCTAGTATTTGATCCCCAT3’ | 60 | 223 |
| hsa_circRNA_103741 | F:5’ACGATGCTTCATCCAGGTCT3’  R:5’ATACACAAGGAGGAACAAACACT3’ | 60 | 203 |
| hsa_circRNA_104096 | F:5’ACATTTGTGATGATAGCCAGTCC 3’  R:5’AGGGTGCTCAGGTTGATTTTG3’ | 60 | 78 |
| hsa_circRNA_101318 | F:5’AGTGTCGTCACCGTAGCCGTA3’  R:5’CTTCCAGCATGGCCTCAATC3’ | 60 | 168 |
| **Housekeeping gene** |  |  |  |
| GAPDH(HUMAN) | F:5’GGGAAACTGTGGCGTGAT3’  R:5’GAGTGGGTGTCGCTGTTGA3’ | 60 | 299 |
