# Supplementary material for: Comprehensive circular RNA profiling reveals that circular RNA100783 is involved in chronic CD28-associated CD8(+)T cell ageing
Source: Immun Ageing. 2015 Oct 8;12:17. doi: 10.1186/s12979-015-0042-z (PMC4597608; doi:10.1186/s12979-015-0042-z)
Supplement: Additional file 3: Figure S1. — Top-5 circRNA-miRNA networks using miRNAs with the 5 highest degrees. Figure S2. Distributions of miRNAs of Top10% Degrees in the Top-5 networks. Figure S3. Re-enriched networks using up-regulated miRNAs (left) and down-regulated miRNAs(right) of Top-10% highest Degree in the original Top-5 network. Figure S4. Distributions of Degree of the miRNAs in the re-enriched Top-10%-mi network. Figure S5. Distribution of Degree of the circRNAs in the re-enriched Top10%-ci network. Figure S6A. Venn’s diagram for optimized circRNA candidates between four groups. Figure S7. The screenshot of the online annotation for the targeted genes in the circRNA100783-targeted miRNA-gene network determined by DAVID annotation. (DOCX 1500 kb) [file 12979_2015_42_MOESM3_ESM.docx]

**Additional file 3**

| 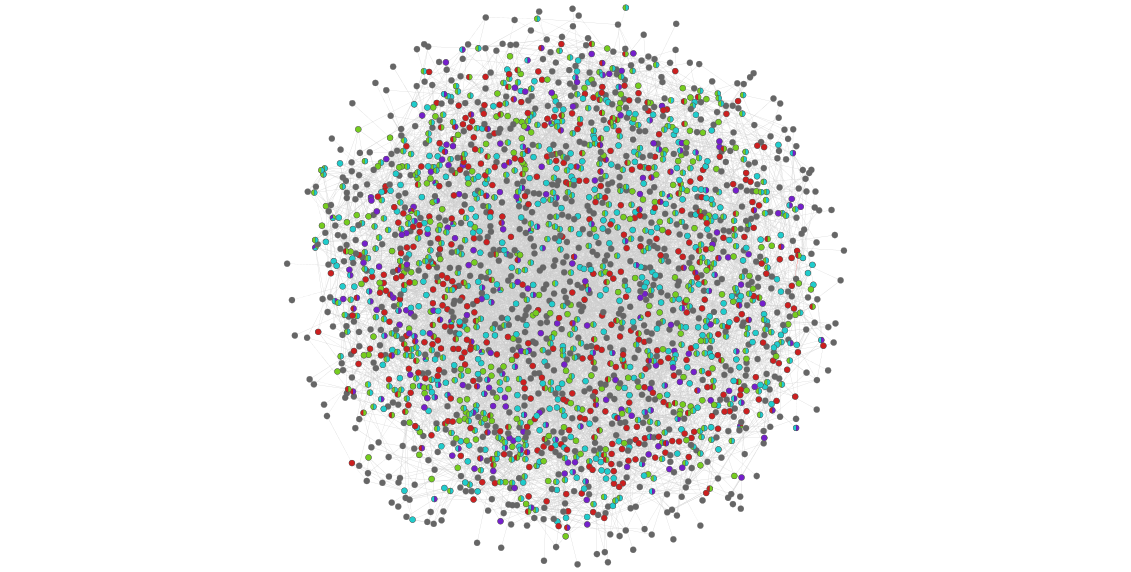left | 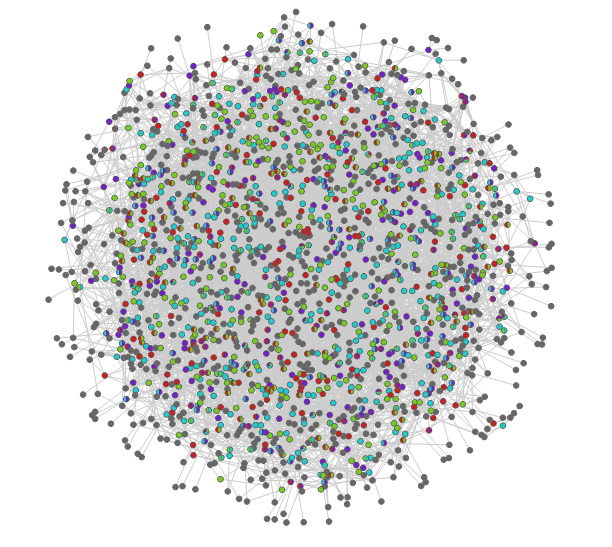right |
| --- | --- |

Figure 1S. Top-5 circRNA-miRNA networks using miRNAs with the 5 highest degrees

We constructed Top-5 networks using Top-5 miRNAs ranking by mirSVR. The up-regulated Top-5 network is comprised of 1865 nodes and 5558 edges(left). The down-regulated Top-5 network is comprised of 2338 nodes and 7723 edges(right). The size of the grey circles represents the Degress of miRNA, while the size of the colored circles represent the Degree of circRNA.


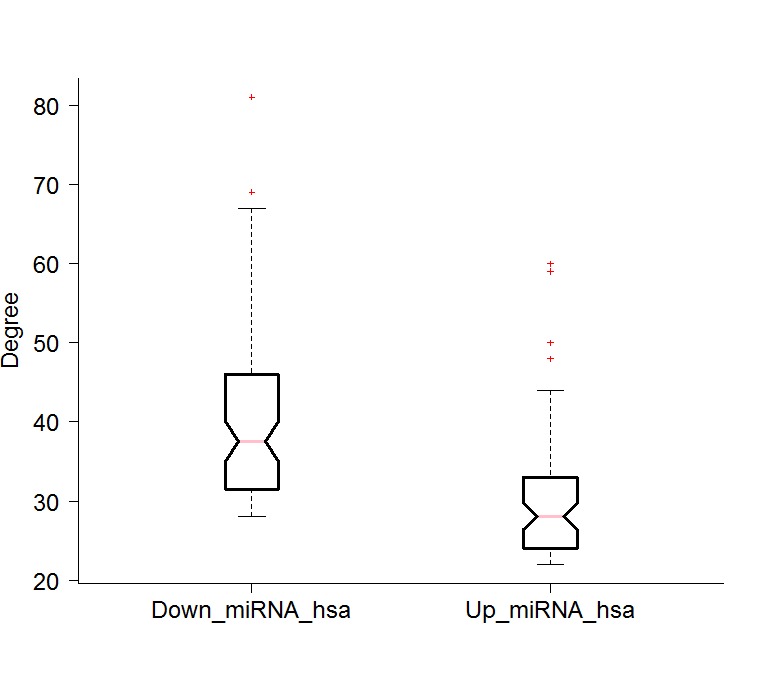


Figure 2S. Distributions of miRNAs of Top10% Degrees in the Top-5 networks

The distribution of Top10% miRNAs optimized from the Top-5 network was depicted by Figure 2S. The Degree indicates the number of miRNAs connected to each circRNA in two Top-5 networks, respectively. The box represents the interquartile range with median value in the middle (pink bar). Two short black bars represent the upper whisker and lower whisker. Red plus signs represent possible outliers.

| 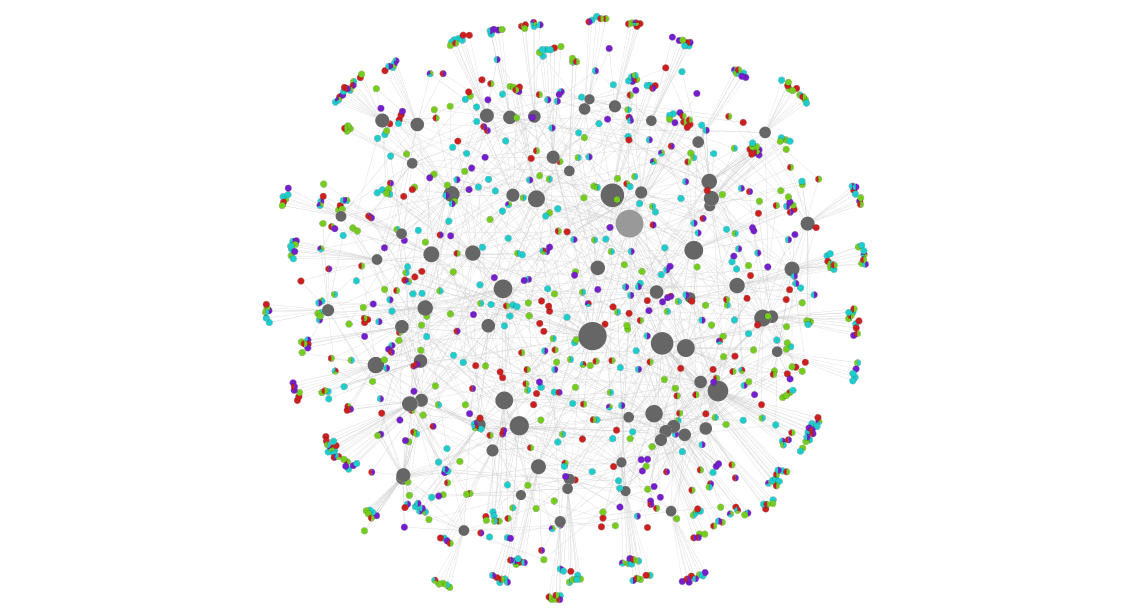left | 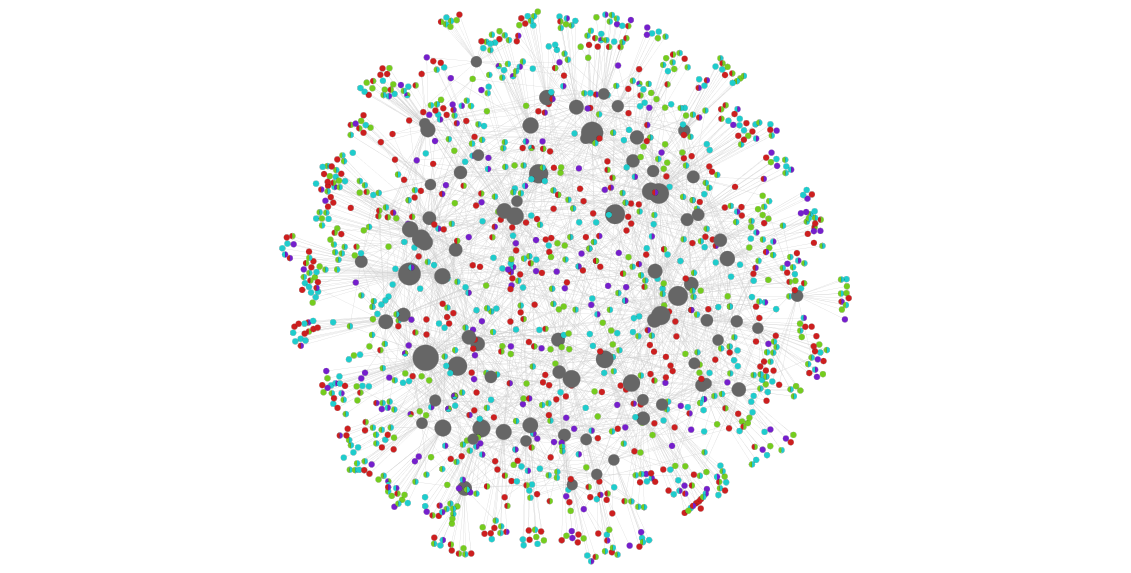right |
| --- | --- |

Figure 3S.Re-enriched networks using up-regulated miRNAs (left) and down-regulated miRNAs(right) of Top-10% highest Degree in the original Top-5 network .

The networks are re-enriched with up-regulated (left) and down-regulated (right) miRNAs of Top-10% highest Degree optimized from the original Top-5 network. The up-regulated network is comprised of 934 nodes and 1591 edges, accordingly, the size of the grey circle which represented the Degree of miRNA ranged from 22 to 60. The down-regulated network is comprised of 1287 nodes and 2261 edges, accordingly, the size of the grey circle ranged from 28 to 81.


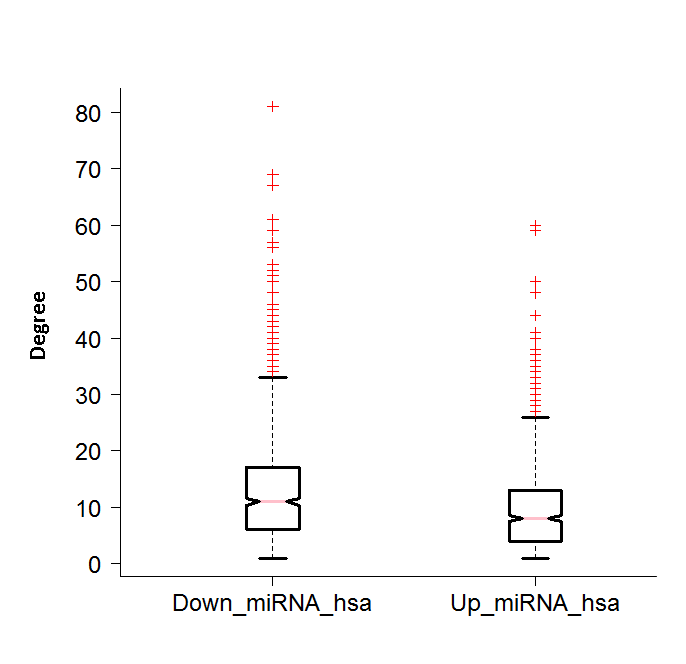


Figure 4S. Distributions of Degree of the miRNAs in the re-enriched Top-10%-mi network

The Degree indicates the number of miRNAs being connected to circRNAs in the re-enriched Top10%-mi networks. Notably, the maximum degree of either up-regulated or down-regulated miRNA in the re-enriched Top-10% network remained the same to that in the original Top-5 networks (D=60 for the up-regulated network and D=81 for the down-regulated network).


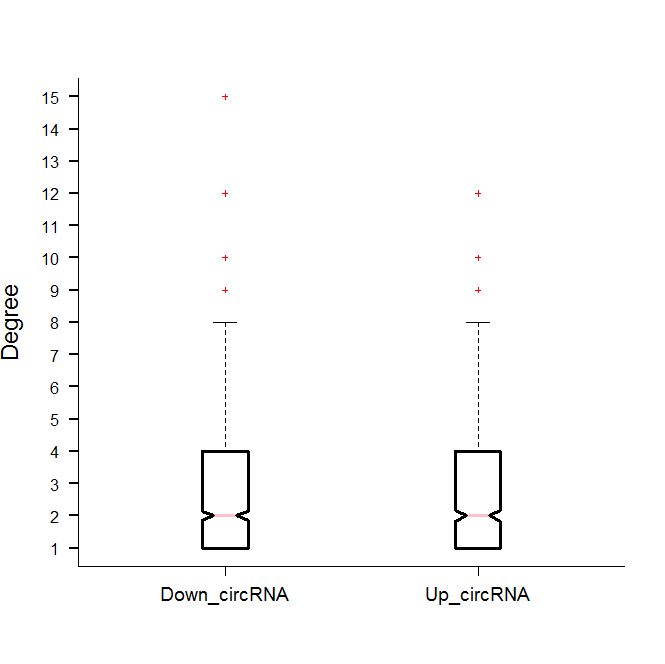


Figure 5S. Distribution of Degree of the circRNAs in the re-enriched Top10%-ci network

The Degree indicates the number of circRNAs being connected to miRNAs in the re-enriched Top-10%-ci networks. Degree of circRNA-based connectivity ranged from 1 to 12 in the up-regulated network and it ranged from 1 to 15 in the down-regulated network.


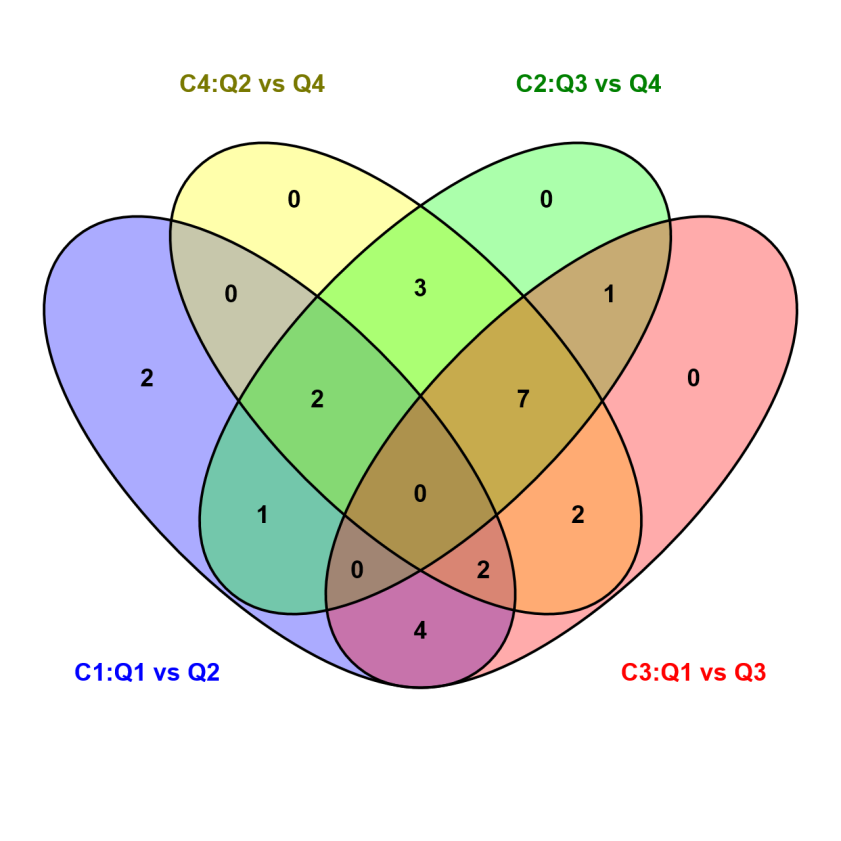


*Color annotation: Comparison 1(C1): blue; Comparison 2(C2): green; Comparison 3 (C3): red; Comparison 4(C4): yellow.

Figure 6S: A Venn’s diagram for optimized circRNA candidates between four groups.


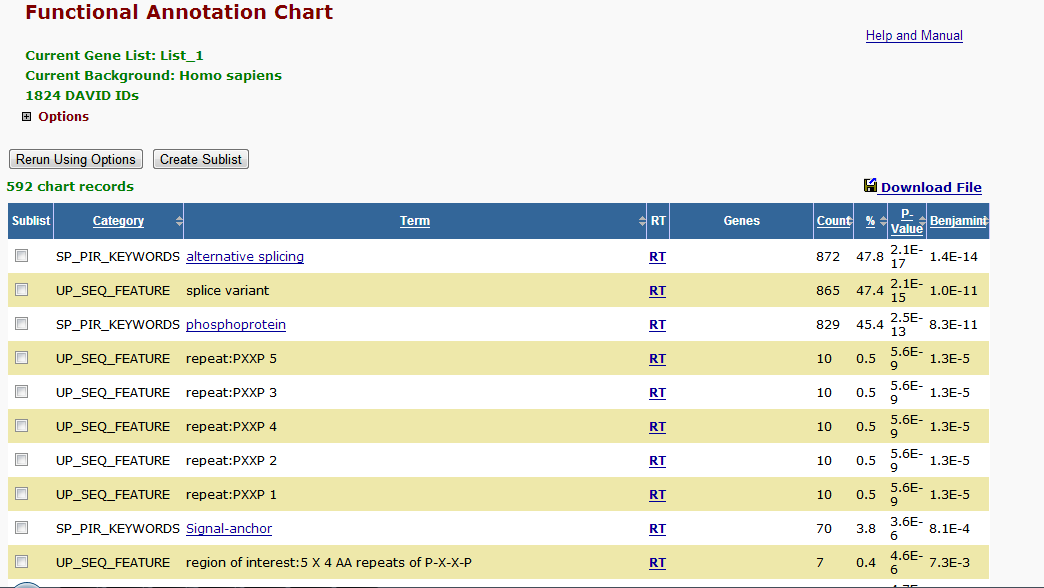


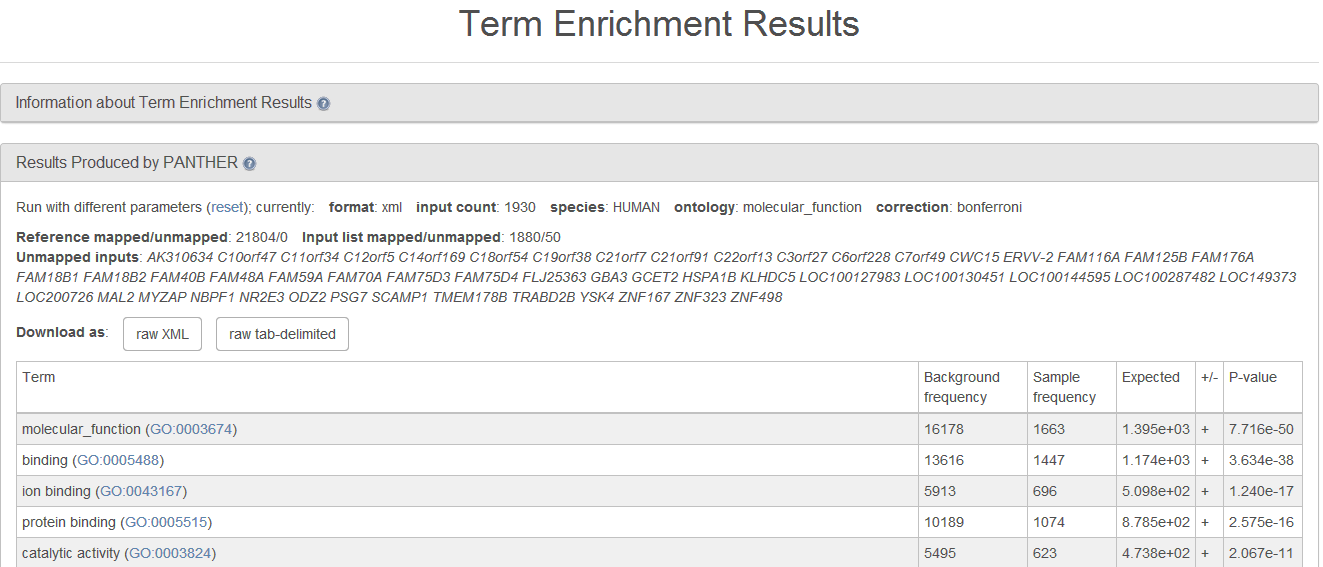


Figure 7S. The screenshot of the online annotation for the targeted genes in the circRNA100783-targeted miRNA-gene network determined by DAVID annotation.
