# Supplementary material for: Comprehensive circular RNA profiling reveals that circular RNA100783 is involved in chronic CD28-associated CD8(+)T cell ageing
Source: Immun Ageing. 2015 Oct 8;12:17. doi: 10.1186/s12979-015-0042-z (PMC4597608; doi:10.1186/s12979-015-0042-z)
Supplement: Additional file 4: — Illustration of step-wised optimization on overlapping circRNAs between C1 and C4. (PPTX 66 kb) [file 12979_2015_42_MOESM4_ESM.pptx]

## Slide 1
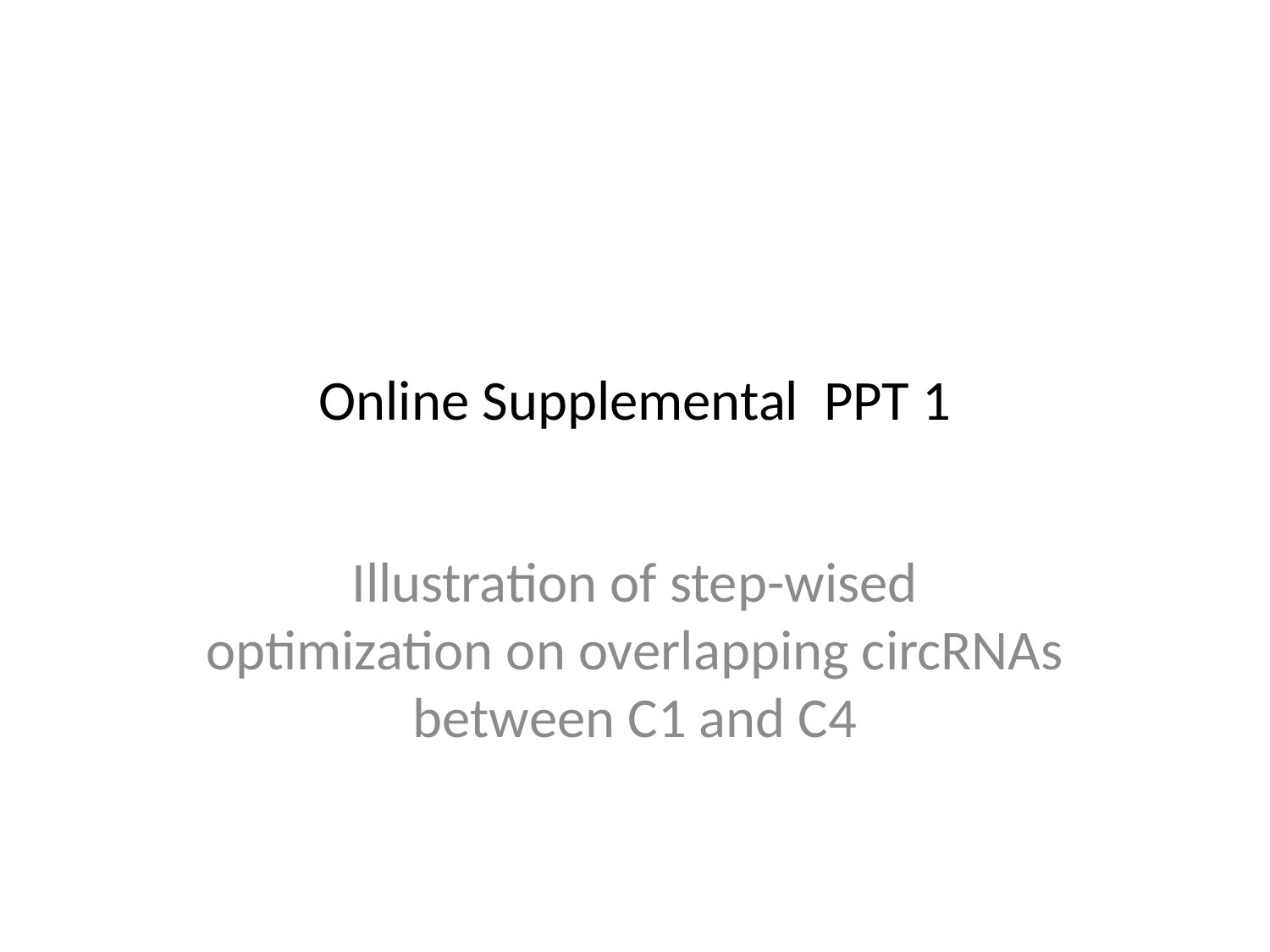

# Online Supplemental PPT 1
Illustration of step-wised optimization on overlapping circRNAs between C1 and C4

## Slide 2
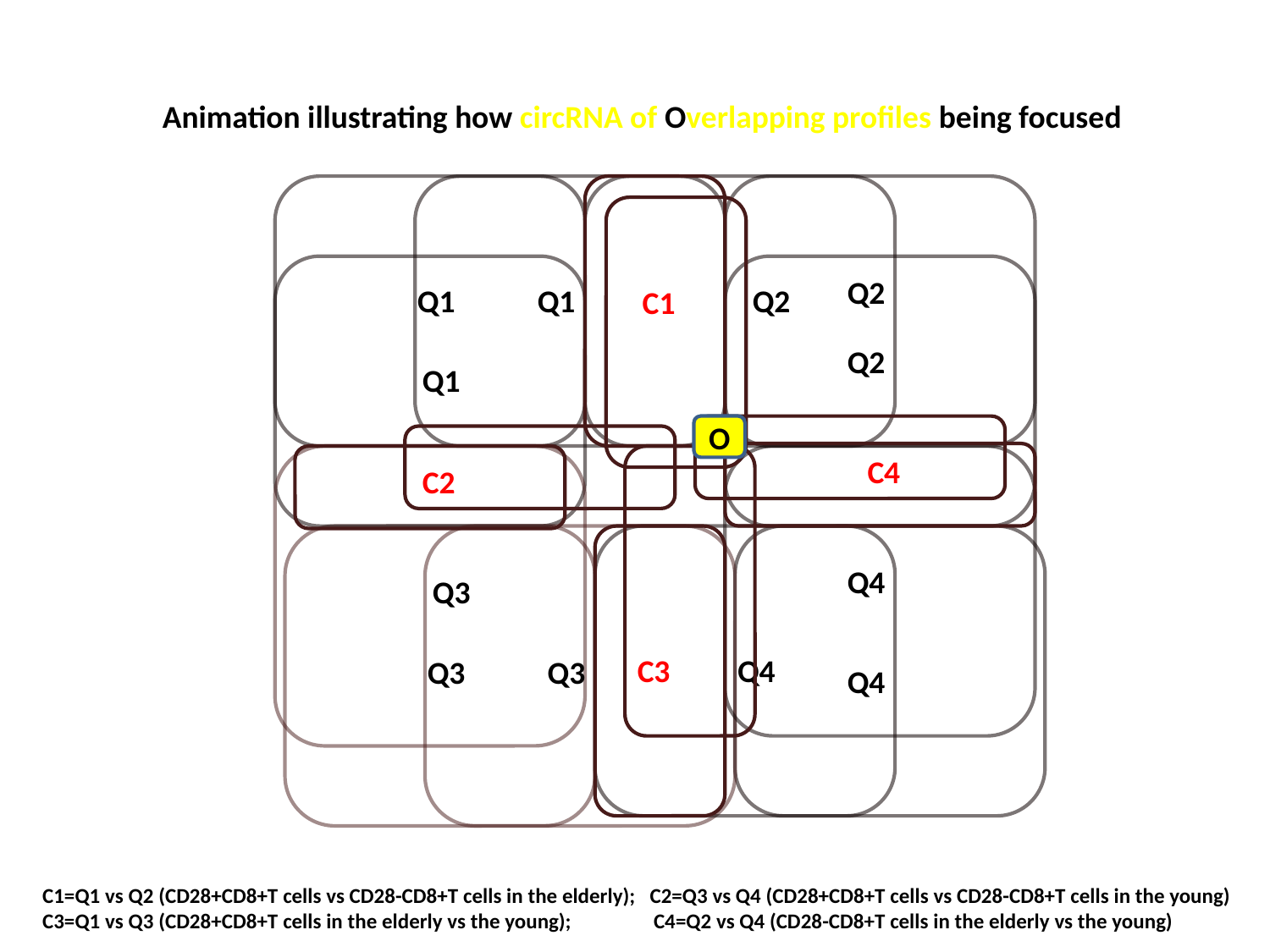

Animation illustrating how circRNA of Overlapping profiles being focused
Q1
Q1
Q2
Q2
Q1
Q2
C1
O
Q3
Q4
C4
C2
Q3
Q3
Q4
Q4
C3
C1=Q1 vs Q2 (CD28+CD8+T cells vs CD28-CD8+T cells in the elderly); C2=Q3 vs Q4 (CD28+CD8+T cells vs CD28-CD8+T cells in the young)
C3=Q1 vs Q3 (CD28+CD8+T cells in the elderly vs the young); C4=Q2 vs Q4 (CD28-CD8+T cells in the elderly vs the young)

## Slide 3
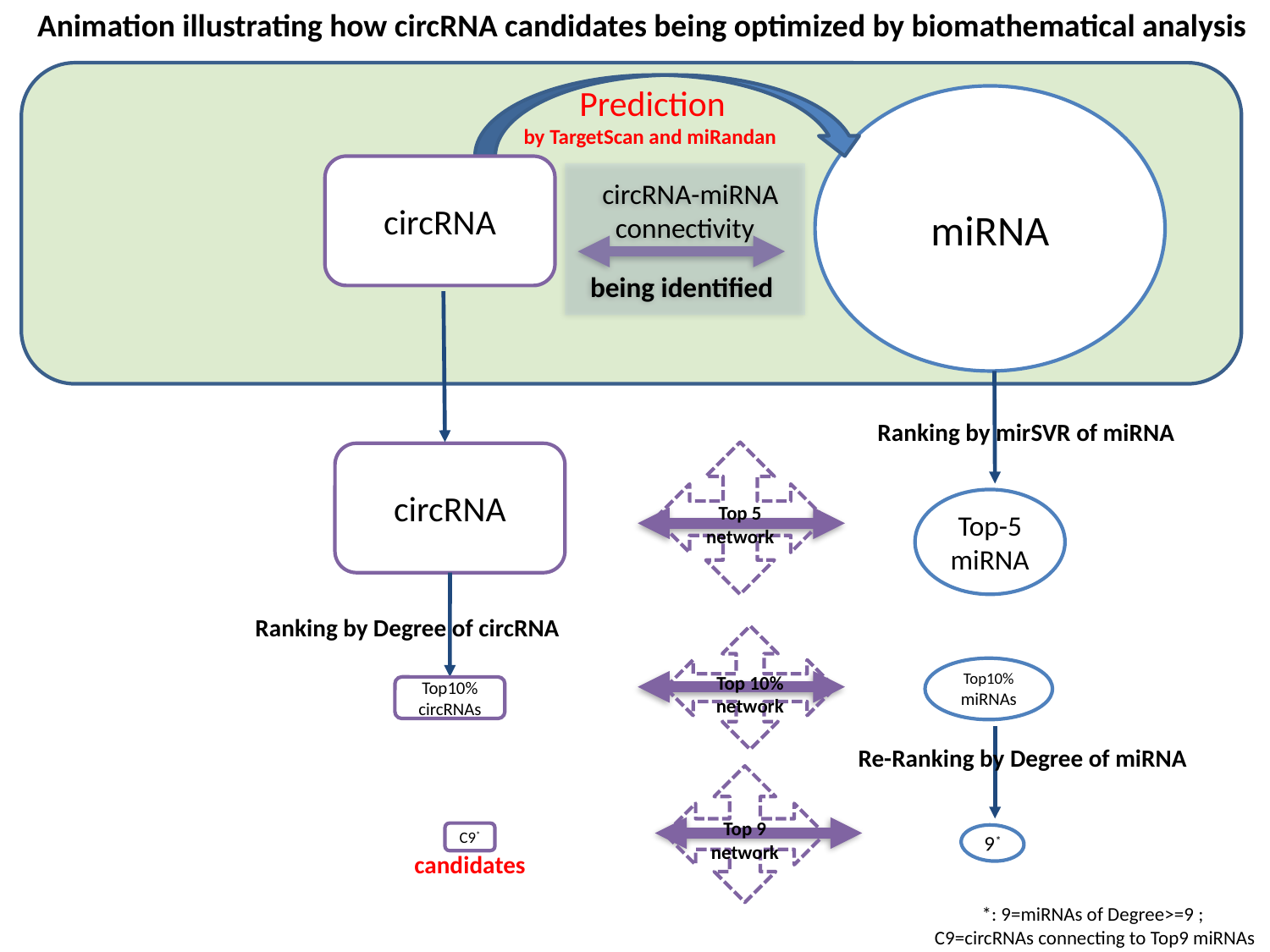

Animation illustrating how circRNA candidates being optimized by biomathematical analysis
Prediction
by TargetScan and miRandan
miRNA
circRNA
 circRNA-miRNA connectivity
being identified
Ranking by mirSVR of miRNA
Top 5
network
circRNA
Top-5 miRNA
Ranking by Degree of circRNA
Top 10%
network
Top10%
miRNAs
Top10%
circRNAs
Re-Ranking by Degree of miRNA
Top 9
network
C9*
9*
candidates
*: 9=miRNAs of Degree>=9 ;
C9=circRNAs connecting to Top9 miRNAs
